# Wine Bottle Refinement: A Review of Emerging Aging Strategies

**DOI:** 10.3390/foods15071269

**Published:** 2026-04-07

**Authors:** Nicola Mercanti, Gregori Lanza, Nathalie Pouzalgues, Monica Macaluso, Fabrizio Palla, Piero Giorgio Verdini, Angela Zinnai

**Affiliations:** 1 Department of Agriculture, Food and Environment, University of Pisa, Via del Borghetto 80, 56124 Pisa, Italy; nicola.mercanti@phd.unipi.it (N.M.); angela.zinnai@unipi.it (A.Z.); 2 Centre du Rosé, 70 Avenue du Président Wilson, 83550 Vidauban, France; gregori.lanza@vignevin.com (G.L.); npouzalgues@centredurose.fr (N.P.); 3 INFNPisa Section, Largo Bruno Pontecorvo 3, 56127 Pisa, Italy; fabrizio.palla@cern.ch (F.P.); piero.giorgio.verdini@cern.ch (P.G.V.); 4 European Organization for Nuclear Research, Espl. des Particules 1, 1211 Meyrin, Switzerland; 5 Interdepartmental Research Centre “Nutraceuticals and Food for Health”, University of Pisa, Via del Borghetto 80, 56124 Pisa, Italy

**Keywords:** oxygen management, underwater aging, wine maturation

## Abstract

Wine bottle aging is governed by complex redox reactions involving phenolic compounds, oxygen transfer and storage conditions, which collectively determine the evolution of wine composition and sensory properties. This review critically examines the main oxidative mechanisms responsible for bottle aging and evaluates traditional and emerging strategies aimed at modulating the evolution of wine. Particular attention is paid to oxygen management, cork type, temperature and light exposure, as well as alternative approaches such as accelerated aging techniques and underwater storage. The available evidence suggests that most accelerated aging technologies fail to replicate the chemical pathways of natural in-bottle aging, often resulting in different aromatic profiles. Attention is paid to underwater aging, an emerging practice that combines specific conditions of temperature, light and limited oxygen availability. The results of the available studies indicate that underwater aging does not significantly alter the basic chemical parameters of wine, but can modulate its phenolic, chromatic and sensory evolution, suggesting a slowdown in oxidative processes compared to traditional aging in the cellar.

## 1. Review Methodology

This review was conducted through a structured literature search aimed at identifying studies addressing the mechanisms of wine aging in the bottle and emerging aging strategies, including underwater storage. The literature search was carried out using the Web of Science, Scopus and PubMed databases.

The following keywords were used, both individually and in combination: “wine aging in bottle”, “wine oxidation”, “wine storage”, “oxygen management in wine”, “accelerated wine aging”, “underwater wine aging”, “wine maturation”, “phenolic evolution of wine” and “wine storage conditions”.

The search covered studies published between 2000 and 2026, with a particular focus on recent publications from 2015 onwards. Only peer-reviewed articles written in English and focusing on the mechanisms of wine aging in the bottle or on alternative aging strategies were included. Studies dealing exclusively with barrel aging, fermentation processes, viticultural practices unrelated to bottle aging, or matrices other than wine were excluded.

## 2. Introduction

Wine is a unique natural product, deeply rooted in the history and culture of numerous civilisations, and represents one of the oldest products obtained from the processing of agricultural raw materials. Its production and consumption have accompanied mankind since ancient times, assuming not only a nutritional value over time, but also a symbolic, social and economic one. Today, the success of wine in the global industry is determined not only by tradition and territorial typicality, but also by continuous scientific and technological advances, which have contributed to improving the quality, safety and standardization of the product [1].

From a regulatory point of view, wine is defined by Regulation (EC) No 1308/2013 as a *“product obtained exclusively from the total or partial alcoholic fermentation of fresh grapes, whether or not crushed, or grape mus”* [2]. This definition emphasizes the indissoluble link between wine and its raw material of origin, highlighting how the fermentation process is the central stage in the transformation of grapes into wine.

### 2.1. Grapevines

Wine is mainly produced from grapes of *Vitis vinifera* L., the species most widely cultivated for winemaking [3]. The grape variety, together with environmental and agronomic conditions, strongly influences must composition and, consequently, the chemical and sensory evolution of wine during storage. Since bottle aging is closely linked to the initial phenolic, acidic and aromatic profile of the wine, varietal composition remains a relevant factor in aging potential.

### 2.2. Wine Production Process

**Figure 1:**
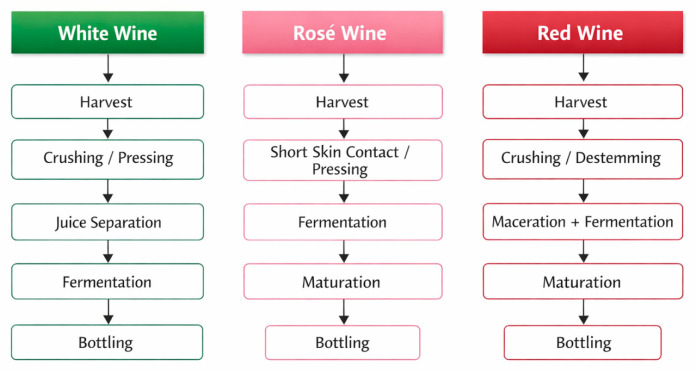
Simplified flowchart of the main winemaking pathways for white, rosé and red wines, highlighting the technological differences relevant to bottle aging.

The production of wine, usually called winemaking or vinification, is a complex procedure (Figure 1), involving different stages, which begins with the selection of the grapes and ends with the bottling of produced wine [4,5,6,7,8].

The first step of the vinification is represented by the harvest [9]. Aromatic maturity specifically encompasses the development of volatile compounds that define a wine’s aroma and is considered a critical factor impacting red wine typicity [9]; technological maturity refers to the sugar and acid balance in grapes, essential for determining harvesting time. This maturity type is generally assessed through Brix measurements (sugar content), as well as acidity levels [10]; phenolic maturity is also significant, influencing color, mouthfeel, and the potential health benefits of wines due to the presence of phenolic compounds, such as tannins and anthocyanins [11]. The right selection and cleaning of the grapes, as well as their integrity, are the first relevant factors for obtaining a high-quality final product [12]. The fruits are then subjected to the mashing phase, during which the grapes are pressed to produce the mash, and to the destemming process, necessary to remove the stalks [13]. At this stage, the first differences between red and white winemaking occur. For white wines, the mash is subjected to draining, aiming at removing the grape skins from the juice, which is then sent to the fermentation process. Red wines, instead, require maceration of the mash with skins and grape seeds, before being sent to fermentation. Once the must has been obtained, it could be advisable to send it to alcoholic fermentation within 24–36 h. Otherwise, the protection of grape juice with the addition of sulphites is strongly recommended [14].

In the winemaking process for rosé wines, the process is halfway between white and red winemaking [15]. Rosé wines are made exclusively from red grapes, but their characteristic color comes from limited contact between the must and the skins, sufficient to allow partial extraction of the anthocyanins responsible for the pink color, but not of tannins in large quantities [16].

The key difference between rosé wine and red wine concerns the timing and duration of maceration. On the one hand, the timing of skin maceration determines the nature of the compounds extracted. In the case of rosé wine, maceration takes place in the must before fermentation, while for red wine it takes place before, during and after fermentation. In other words, the diffusion of compounds from the skins occurs in an aqueous phase for rosé wine, but in the presence of alcohol for red wine. It is highly likely that the molecules extracted are not the same in the two cases [15]. On the other hand, the duration of maceration influences the quantity of compounds extracted: in the case of rosé, skin maceration varies from a few minutes to a few hours, while for red wine it varies from a few days to a few weeks [15]. The essential difference between rosé wine and white wine concerns the nature of the grape varieties, since, in fact, the viticulture and vinification of rosé wines are similar to those of white wines [15].

From a fermentation point of view, rosé wines are generally fermented at temperatures similar to those of white wines, between 10 and 18 °C, in order to preserve their fresh and fruity aromas. Limited phenolic extraction gives rosé wines a lighter structure than red wines and greater freshness, making them products characterized by a balance between aroma, acidity and color.

There are three main methods of producing rosé wines: direct pressing, bleeding and maceration [17]. In direct pressing, the grapes, harvested by hand or mechanically, are taken directly to the press, where the juice flows continuously. In general, direct pressing produces a lightly colored and rather acidic must. In the case of bleeding, the grapes are placed in tanks after destemming and crushing, where they are left to macerate for several hours (5 to 24), preventing fermentation from starting. When the desired color is obtained, part of the juice (5 to 15%) is drained off. This is known as “bleeding” the vats. This juice will form the basis of the rosé wines.

The rest of the tank allows the production of a red wine rich in polyphenols. Unlike direct pressing, bleeding ensures significant diffusion of polyphenols and gives the must characteristics more similar to those of red wines, both in terms of color and aroma. Finally, the third production technique is maceration. Intermediate between the first two methods, maceration in vats or closed cage presses ensures controlled contact between the juice and the skins, preventing fermentation from starting. However, unlike bleeding, after all the juice has been decanted, the fresh pomace is pressed and can be reincorporated in whole or in part into the free-run juice. This latter technique, known as maceration, allows for considerable diversity depending on the duration and temperature conditions. The management of maceration has a significant influence on the color of the wine. It also has consequences on oenological and organoleptic parameters other than appearance [18]. Two factors have been studied at the Centre du Rosé: the temperature and duration of maceration. Both allow the contact between the juice and the solid matter to be modulated [17]. Foreign studies, such as the Italian study on a specific grape variety, Bombino Nero, and on the contact time between the juice and the grape skins, have demonstrated the importance of this parameter in the production of rosé wines and complement the work of the Centre du Rosé [17].

The primary objective of fermentation is the conversion of sugars into ethanol and carbon dioxide, even though the formation of other secondary volatile and non-volatile secondary metabolites, responsible for the wine features, is also fundamental. The fermentation of the must is determined by different yeast strains, both autochthonous naturally occurring on the grapevines and selected, usually belonging to *Saccharomyces cerevisiae* strains [14,19]. In the earliest stages of fermentation, also called pre-fermentation, the yeasts carry out aerobic respiration, transforming the sugars into water and carbon dioxide. Once the oxygen is finished, the real fermentation phase takes place, during which the yeasts switch to anaerobic metabolism, and convert almost 91% of the sugars into ethanol, through alcoholic fermentation. The rest of the sugars, instead, undergo glycerol–pyruvic fermentation, responsible for the production of small amounts of secondary products, which are therefore of great importance from the organoleptic point of view [14]. The fermentation process is an exothermic process that creates heat naturally. During the red vinification, fermentation starts at about 20 °C, and the heat production may lead the temperature to rise to 30–32 °C. Yeasts are susceptible to temperatures above 35 °C, and thus, to reach the whole sugar fermentation, it is important to maintain the must temperature below this level, using some kinds of control [8]. During fermentation, the production of CO_2_ causes the solid skins and grapeseed to rise to the liquid surface, creating a floating cap. Since the skins are required to be in contact with the juice in order to have a good extraction, a process called remontage is performed: the juice is taken from the bottom of the vat, pumped up, and sprayed on the cap surface, to submerge it, increasing their contact with the juice and also boosting the yeasts. At this point, depending on the style, the wine can be left to macerate with skins for a period of time variable from a few days to 28 days, to achieve a good extraction of pigments and tannins and a good formation of flavors. The wine is then transferred to another vessel, leaving the sediments in the previous vats, through the operation of racking. The skins, instead, are subjected to pressing, which makes it possible to obtain further juices rich in tannins and pigments.

For white wines, as previously mentioned, the skins are not employed during the fermentation, but are separated from the juice through the draining process, and then subjected to a gentle pressing. The obtained juice should be as clear as possible, since solids can determine off-tastes: for this reason, a clarification of 12–24, called débourbage, can be performed. The must is then passed through a heat exchanger to decrease the temperature, preventing the premature fermentation as well as maintaining the freshness and the flavors, and is added with sulphites and eventually with yeasts, before being sent to the fermentation vessel. White wines are usually fermented at lower temperatures, about 10–18 °C, and for longer periods than red ones. Cooler temperatures are advisable to preserve the aromatic fruit flavors. Some white wines are fermented in barrels to give particular characteristics to the wines and a better integration of oak flavors [8].

After alcoholic fermentation, all red wines and the well structured white wines undergo malolactic fermentation, which consists of the transformation of malic acid in lactic acid, carried out by lactic bacteria of the genera *Lactobacillus*, *Leuconostoc*, and *Pediococcus*. This fermentation determines the elimination of the sour taste caused by malic acid and the increase in lactic acid, responsible for a smooth and round taste, due in turn to the reduced fixed acidity. In white wines, where acidity is valued, malolactic fermentation is not recommended [8,14]. Once fermentation is complete, the blending of the single wines, obtained from the separated fermentation of different grape varieties, is performed to achieve the desired final style and quality of the product.

The wines then undergo a period of maturation, during which the tannins soften and acidity decreases. The type of vats for maturation and the period depend on the desired style. Stainless steel is an ideal storage material as it is impermeable to gases such as oxygen. Most high-quality red wines undergo a period of barrel maturation, usually comprised between 9 and 22 months, during which the wines absorb some oak products, including wood tannins and vanillin, while most white wines are stored in stainless steel or concrete vats until ready for bottling, or they can continue their maturation in the barrel to obtain more oak flavors [8]. After maturation, several treatments may be carried out to ensure the wine’s final stability. These include fining, filtration, and other procedures, after which the wine is bottled.

### 2.3. Types of Wines

The significant differences between the typical production of white, red, and rosé wines can be briefly summarized as follows:

• White grapes are crushed/destemmed and pressed to juice and do not typically spend much time in contact with grape solids (there is no maceration step).
• Red grapes are crushed/destemmed and the must (juice and grape solids) undergoes maceration and fermentation in the presence of skins, seeds and juice.
• Rosé wines: Rosé wines are typically produced from red grapes, but these are pressed or the must remains in contact with the skins for only a short period, resulting in limited color extraction before pressing and fermentation, which usually proceeds without the skins.

The production techniques for the different types of wine are shown in the following table (Table 1).

As a result of these differences, polyphenol extraction is minimized in white wine production, reduced in rosé wine production and intentionally favored in red wine production [21,22].

White and rosé wine fermentation is usually carried out at lower temperatures to preserve aromatic compounds, while red wine fermentation uses higher temperatures to promote the extraction of phenolic compounds from the solid parts of the grapes [20].

Exposure to oxygen is generally limited during the fermentation of white and rosé wine, while in red wine vinification, controlled oxygenation is commonly applied as part of maceration practices.

Most red wines undergo malolactic fermentation (MLF) and oak aging, unlike white wines, for which these treatments are limited to specific styles [20,20,23].

As a result, white and rosé wines can often be released to the market earlier than red wines, which typically benefit from a longer aging period before commercialisation.

### 2.4. Wine Oxidation

Wine oxidation refers to a complex series of oxygen-triggered chemical reactions that affect the composition, stability and sensory properties of wine [24]. Oxidation can be beneficial or detrimental depending on the degree of exposure to oxygen, the composition of the wine matrix and the timing of its occurrence during production or aging [25,26].

Molecular oxygen (O_2_) is relatively unreactive with most wine components [24]. Oxidation therefore proceeds through a gradual reduction of oxygen, forming increasingly reactive intermediates [27,28,29]. The overall reduction of oxygen can be summarized as follows:

$$O_{2}+4e^{-}+4H^{+}\to 2H_{2}O$$

In wine, this reduction occurs through multiple one-electron steps, producing reactive oxygen species (ROS) that drive oxidation reactions.

The first step of wine oxidation involves the reduction of oxygen to superoxide and hydrogen peroxide, typically catalyzed by phenolic compounds and metal ions:

$$O_{2}+e^{-}\to O_{2}^{.-}$$

$$2O_{2}^{.-}+2H^{+}\to H_{2}O_{2}+O_{2}$$

Hydrogen peroxide (H_2_O_2_) is a key intermediate because, while not highly reactive itself, it acts as a precursor to more aggressive oxidizing species [25,30,31].

Phenolic compounds, particularly o-diphenols, are central substrates in wine oxidation [20]. In the presence of oxygen and metal catalysts, phenols are oxidized to quinones:

$$Phenol\to Quinone+2e^{-}+2H^{+}$$

Quinones are highly electrophilic and chemically unstable. Unlike oxygen, they readily react with nucleophilic wine constituents, making them critical intermediates in oxidative pathways [23,32].

Once formed, quinones undergo secondary reactions that profoundly affect wine composition [20]. Major nucleophilic targets include the following:

#### 2.4.1. Sulphur-Containing Compounds

Thiols (R–SH), responsible for key varietal aromas in white wines, readily react with quinones:

Quinone+RSH→Phenol-SR

This reaction results in the irreversible loss of aroma compounds [33].

#### 2.4.2. Sulphur Dioxide

Sulphur dioxide reacts with quinones, regenerating the reduced phenolic form:

$$Quinone+HSO_{3}^{-}\to Phenol\text{-}SO_{3}^{-}$$

This reaction underpins the antioxidant role of SO_2_ in wine [34,35].

#### 2.4.3. Phenolic Coupling

Quinones can also react with other phenolics, leading to polymerization reactions that contribute to the following:

• Color stabilization;
• Tannin evolution;
• Changes in mouthfeel.

Oxidative behavior varies significantly among wine styles:

• White wines are highly susceptible to oxidation due to their low phenolic content and rely heavily on SO_2_ protection and reductive handling.
• Red wines possess greater oxidative buffering capacity because of higher phenolic concentrations and often benefit from controlled oxygen exposure.
• Rosé wines represent an intermediate case, requiring careful oxygen management to preserve color and aroma.

Although the main oxidative pathways of wine are well established, direct comparison among studies remains difficult because oxidation kinetics are strongly influenced by the wine matrix, phenolic composition, metal content, sulphur dioxide concentration and storage conditions. As a result, the relative contribution of the different oxidative mechanisms may vary substantially among wine styles, and conclusions derived from one matrix cannot always be directly transferred to another. This variability highlights the need for more standardized comparative studies, particularly when evaluating bottle aging under different oxygen management conditions.

### 2.5. Specificity of the Oxidative Stability of Rosé Wines

Due to their chemical nature (polyphenols, aromas), rosé wines are considered fragile and special care must be taken to obtain the desired color and aromatic profile. In fact, the substances most involved in oxidation reactions are polyphenols [36], and the ability of wines to consume oxygen is directly related to their quantity [37]. The low polyphenol content of rosé wines explains why, at room temperature, the oxygen consumption time in most rosés is around 30 days, in line with that of white wines. In fact, in a comparison between red and rosé wines from the same batch of red [38], Grenache showed that tannins are the main phenolic compounds in red wines, while hydroxycinnamic acids are largely predominant in rosés. However, in both rosé and white wines, the oxidation of polyphenols such as flavanols can lead to browning of the must. In the composition of the must in hydroxycinnamic acids, glutathione and polyphenol oxidase determine in particular its browning upon oxidation [39]. Depending on the type of vinification, hyperoxygenation techniques or, conversely, hyperreduction techniques can be used, ranging from the preventive precipitation of oxidisable compounds to their protection by inertisation. Furthermore, an excess of polyphenols can not only affect the desired light color, but also the aromatic quality, particularly because the oxidation of polyphenols can lead to the formation of quinones that can react with thiols and form compounds that are inactive from a sensory point of view [40]. Since thiols are highly oxidisable aromatic compounds, these phenomena have led to rosé winemaking processes geared towards protection from oxidation, particularly through inertisation and the addition of sulphites. However, the stages following vinification remain a crucial point to consider in the overall reflection on improving the stability of rosé and white wines. These stages include both the various practices related to aging and storage.

### 2.6. Physical Factors Influencing Wine Bottle Aging

#### 2.6.1. Type of Closure

Wine aging can also be carried out in glass bottles of various capacities, which typically hold 0.75 L of wine [41]. Thanks to its hermetic nature, in fact, glass allows oxygen to permeate exclusively through the cork [42].

**Figure 2:**
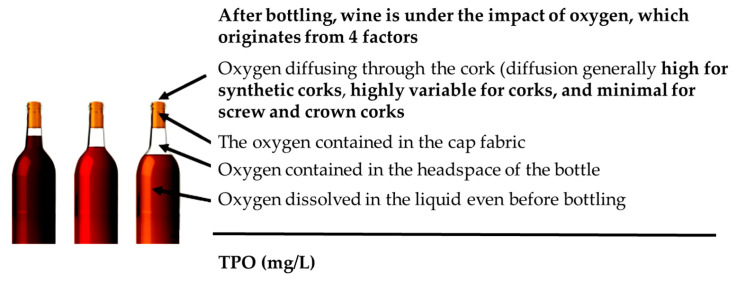
Oxygen input in the bottle (TPO). Reprinted from reference [24].

However, the penetration of the oxygen in the bottle through the stopper could represent an important factor when bottle aging is desired to enhance the flavor and aroma of the wine [26] and the amount of oxygen able to penetrate inside the bottle can be defined as TPO (Total Oxygen Package) [43] (Figure 2).

**Figure 3:**
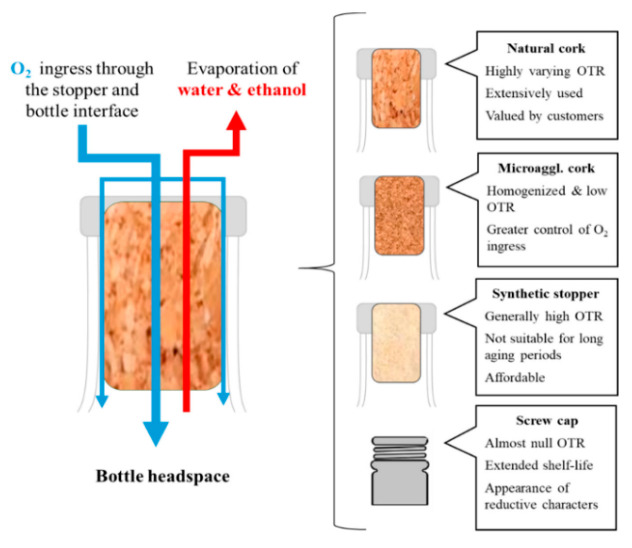
Ingress of the oxygen in the bottle and the characteristics of caps. Reprinted from reference [26].

A great number of studies evidenced that the stopper characteristics also greatly influence the bottle aging process and preservation of the wine [44,45,46], as the type of cap used is able to modulate the oxygen ingress in the bottle (Figure 3) [26].

This influence is mainly related to differences in the oxygen transmission rate (OTR), which affect the extent of oxidative and reductive evolution during bottle storage. Closures with higher oxygen permeability may accelerate the loss of free sulphur dioxide, promote browning and alter the volatile profile, whereas closures with very low oxygen ingress may better preserve freshness but also increase the risk of reductive notes [26]. Therefore, stopper selection directly affects the balance between aroma preservation, color stability and oxidative development during aging.

To this day, there are three main types of corks used during bottling: natural corks (agglomerated or single-piece), synthetic corks, and crown caps [47].

Natural cork can release some volatile compounds, such as alcohols, terpenes, aldehydes, and ketones, which greatly impact the volatile flavor of the wine.

Synthetic closures result in the wine having the highest oxygen transfer rate and the lowest antioxidant levels, and they are commonly associated with an oxidized aroma in white wine [48].

Several studies have shown that synthetic corks can be of great value for young wines and those aged for a short period [49].

Crown caps are characterized by a very low and reproducible oxygen transmission rate (OTR), ensuring excellent sealing and minimal oxygen ingress during bottle aging [50]. This closure type significantly limits oxidative reactions and allows a more controlled and predictable evolution of the wine over time. However, the almost anaerobic environment created by crown caps may promote reductive aromas, such as hydrogen sulfide and other sulphur-containing compounds, particularly in wines with low redox buffering capacity [51].

#### 2.6.2. Storage Time

The duration of bottle aging plays a crucial role in the formation of the wine aroma, as prolonged aging increases the influence of oxidative factors such as the type of cork, temperature and exposure to light, thus accelerating oxidative processes [48]. Excessive oxidation in red and rosé wines can lead to the degradation of key aromatic compounds, resulting in a reduction in aroma and, in extreme cases, deterioration of the wine. White wines are particularly sensitive, often showing browning and a marked decline in varietal aromas. Conversely, some types of intentionally oxidized wine, including Sherry, Madeira and Port, benefit from prolonged storage, during which oxidative reactions contribute positively to the complexity of the aroma.

Several studies report a gradual decline in total sulphur dioxide levels with increasing storage time, with approximately 55% of the initial free SO_2_ lost in the first four months after bottling [52]. This decrease is probably associated with reactions between sulphur dioxide and flavanols during the long bottle aging of red wines [53]. As storage continues, the wine environment becomes increasingly reductive, favoring the accumulation of hydrogen sulphide (H_2_S) [52]. Despite these changes, polyphenol concentrations remain relatively stable after 15 months of bottle aging, largely due to the protective role of SO_2_ [54]. Furthermore, it has been shown that the overall aromatic intensity of rosé and white wines aged for 18 months in the bottle decreases significantly, particularly for descriptors such as “tropical fruit”, “floral”, “wild berries” and “sweet” [55].

#### 2.6.3. Temperature

Temperature is one of the most important factors causing wine deterioration. High temperatures irreversibly alter the chemical and organoleptic characteristics of wine, accelerating the aging process. When the temperature exceeds 25 °C, the freshness of the wine decreases and undesirable properties increase [56]. It has been observed that when wines were stored at 40 °C, the concentration of 1,1,6-trimethyl-1,2-dihydronaphthalene (TDN) norisoprenoids was higher, esters and acetates were reduced, and the aging process was accelerated. This gave the wine aromas of diesel, oxidation and rubber [48].

Temperature also affects the concentration of tannins and anthocyanins.

It has been reported that tannins decrease in Malbec wines during storage. However, at 15 °C, tannins were higher than at 25 °C during a complete aging process; it has been estimated that high temperatures and low pH (of the wine) accelerate the loss of high molecular weight tannins [57].

**Figure 4:**
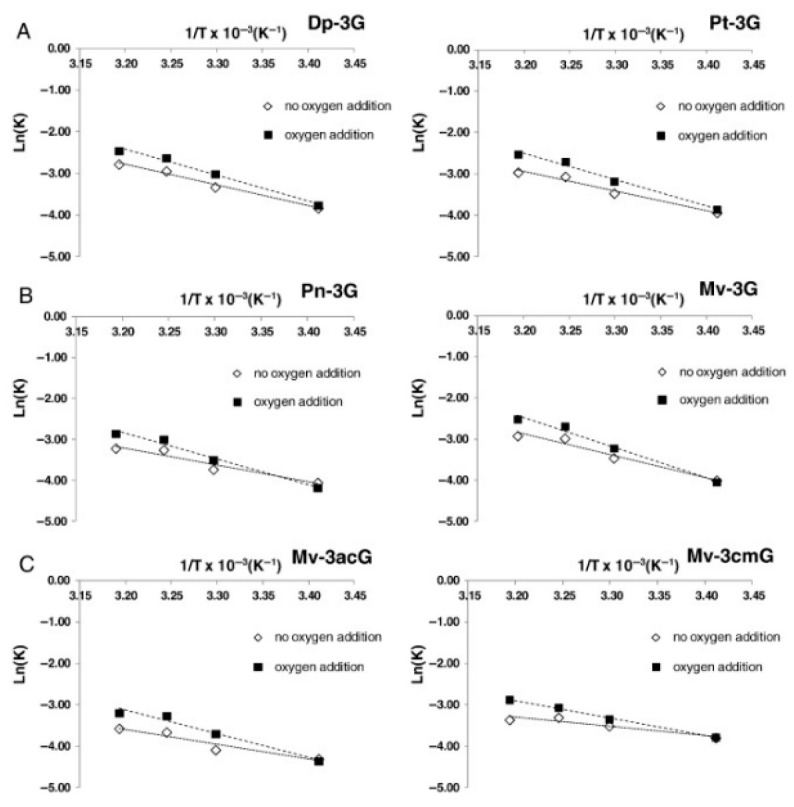
Arrhenius plot for delphinidin-3-glucoside (Dp-3G) and petunidin-3-glucoside (Pt-3G) depletion (**A**); for peonidin-3-glucoside (Pn-3G) and malvidin-3-glucoside (Mv-3G) depletion (**B**); and for malvidin-3-O-acetylglucoside (Mv-3acG) and malvidin-3-O-coumaroylglucoside (Mv-3cmG) depletion (**C**). Reprinted from reference [40].

As shown in Figure 4, increasing temperature accelerates anthocyanin depletion, supporting the view that thermal conditions are a key driver of chromatic instability during bottle aging.

This decline contributes to color instability, loss of chromatic intensity and accelerated aging-related changes in red wines, confirming that temperature control is a critical factor for preserving wine quality during bottle storage.

#### 2.6.4. Light

Exposure to light is one of the main factors determining oxidative processes in bottled wine. Light-induced redox reactions promote the formation of compounds such as acetaldehyde, which contribute to undesirable sensory notes, while reducing key aromatic characteristics, particularly fruity ones. Ultraviolet (UV) radiation has been shown to accelerate the depletion of free sulphur dioxide [58], thereby weakening its protective function against oxidation [59]. Furthermore, exposure to light alters the redox balance of iron by shifting the Fe(III)/Fe(II) ratio towards the reduced form, which enhances Fenton-type reactions [24]. This shift can trigger photo-Fenton mechanisms that further intensify oxidative reactions and accelerate wine degradation during bottle aging [60].

Taken together, these factors show that bottle aging is not determined by time alone, but by the complex interaction between wine composition, oxygen management and storage conditions, which jointly shape the sensory and chemical evolution of wine.

### 2.7. Positive Change in Bottling Aging

The positive changes include the transformation of wine polyphenols, anthocyanins and tannins, as well as the formation of certain thiols and aromatic aldehydes that contribute significantly to the refined aroma and flavor of aged wines [26].

#### 2.7.1. Anthocyanins

Anthocyanins are the main pigments in red grapes and are the compounds responsible for the color of wine. They are formed in the skin of grapes from catechin and epicatechin. A fundamental aspect is that the aging and storage of wine cause a transformation in color from dark shades to bright red, through structural modifications and polymerization processes of these compounds [26].

Anthocyanins can exist in different chemical forms, which are present in varying proportions and in chemical equilibrium with each other [61].

**Figure 5:**
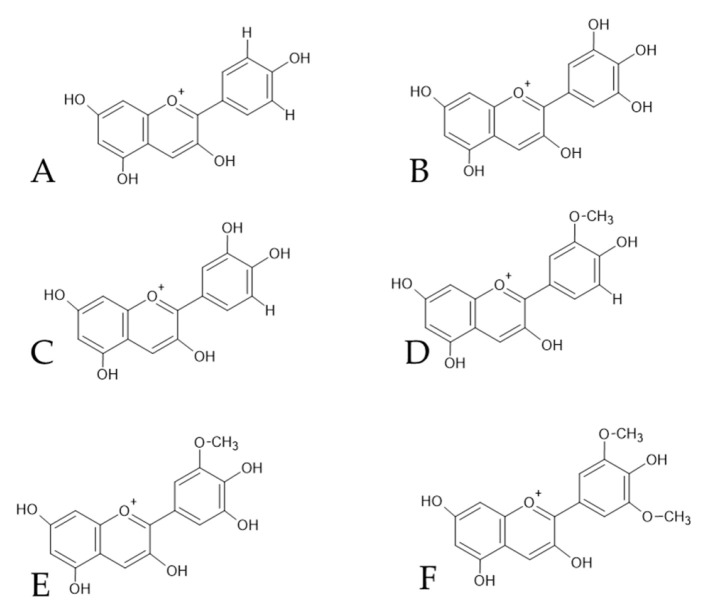
The most relevant type of anthocyanins presents in wine. (**A**): cyanidin, (**B**): delphinidin, (**C**): pelargonidin, (**D**): peonidin, (**E**): petunidin, (**F**): malvidin.

Among the numerous anthocyanins present, the most relevant are delphinidin, cyanidin, petunidin, peonidin, malvidin, and pelargonidin (Figure 5), all derivatives of 3-O-glucosidic anthocyanins [62].

New anthocyanin pigments can originate through various interactions with other molecules in wine, such as aldehydes, acetaldehyde or flavanols (condensed tannins) [63]. In addition, smaller molecules, such as phenolic acids (particularly hydroxycinnamic acids) and phenolic compounds (PCs), such as guaiacol and syringol, act as precursors to the chemical transformations undergone by anthocyanins [26].

These pigments, characterized by different structures, have more intense colors and significantly greater stability against chemical and pH variations in wine, thus helping to make the color of wine much more resistant to possible alterations [64]. A particularly significant effect is the greater resistance of the color to bleaching caused by sulphur dioxide (SO_2_), one of the main properties of this antioxidant [65].

Pigments derived from anthocyanins generally give rise to pyranoanthocyanins. Their name derives from the presence of a pyran ring (D), formed between positions C4 and C5 of the anthocyanidin base unit, from which various radicals can originate and which also acts as a binding site for polymerization [64].

The transformation of pigments derived from wine anthocyanins generally gives red wines a brighter color, due to the presence of vitisins, accompanied by darker chromatic notes attributable to porstisins, pinotins and other adducts. As for white wines, color variations are not related to anthocyanin pigments, but to the formation of pigments and salts derived from xanthyl [66].

Consequently, the color of white wines depends mainly on the formation of these xanthyl salts and condensed tannins and flavonoids, such as (+)-catechin or (−)-epicatechin [26]. In this context, the degradation of flavonoids has been linked to an increase in the browning index, as it promotes the formation of brown pigments.

#### 2.7.2. Aldehydes

Acetaldehyde is the most abundant aldehyde produced as a direct outcome of oxidative chain reactions and represents the predominant aromatic aldehyde found in wine. For this reason, it is widely employed as an indicator of oxidative processes, closely associated with the depletion of SO_2_. From a sensory perspective, acetaldehyde contributes pleasant fruity notes at low concentrations (approximately 30 mg/L), whereas at higher levels (around 100 mg/L) it is responsible for undesirable aromas reminiscent of spoilage [24].

During bottle aging, furan sotolon is formed by condensation of alpha-keto butyric acid and acetaldehyde or by degradation of ascorbic acid by ethanol [48], which has a strong “roast”, “caramel”, and “curry” odor [67].

Acetaldehyde participates extensively in multiple concurrent reactions occurring during bottle aging, thereby playing a central role in wine evolution. At the same time, its concentration must be carefully controlled to prevent oxidative deterioration. Notably, acetaldehyde reacts rapidly with bisulphite ions (SO_3_^−^), forming an insoluble disulphite-bound adduct with reduced aromatic impact [26,68]. Consequently, in wines lacking SO_2_ addition, acetaldehyde is likely to become one of the dominant aroma compounds [69].

In addition to acetaldehyde, several other aldehydes are associated with oxidative conditions and may exert either positive or negative effects on wine aroma depending on whether their concentrations fall below or exceed their sensory perception thresholds. Aldehydes such as octanal, nonanal, and decanal, which are generally regarded as desirable aroma contributors, can generate unpleasant odors when oxidation leads to concentrations well above their perception limits [32].

Conversely, certain aldehydes, including phenylacetaldehyde, are linked to sweet, honey-like aromas that can enhance the overall aromatic profile of wines [70]. Aldehydes originating from oak barrels during oxidative aging, such as furfural, typically exhibit a decreasing concentration over time, as they undergo degradation or react with other wine constituents like quinones. In particular, furfural, considered a key positive aromatic aldehyde, tends to diminish during bottle storage through reactions with other wine components, contributing either to the formation of xanthylium cations or to the generation of aromatic thiols [26].

#### 2.7.3. Other Compounds

Several additional compounds significantly influence the sensory evolution of wine during bottle aging, including tannins, norisoprenoids, terpenols, and thiols. Wine tannins are predominantly condensed tannins (proanthocyanidins) derived from grape seeds and skins [71]. Structurally, they consist of polymers of flavan-3-ols—such as (+)-catechin, (−)-epicatechin, (−)-epigallocatechin, and (−)-epicatechin-3-O-gallate—linked mainly through C4–C6 or C4–C8 bonds [26]. During bottle storage, tannins undergo hydrolytic and oxidative transformations that release reactive flavanols, promote the formation of stable anthocyanin-derived pigments, and gradually reduce astringency and color intensity. Over extended aging periods, tannin degradation tends to dominate over repolymerization, resulting in an overall decrease in tannin levels [72], while excessive polymerization can lead to precipitation and removal through fining treatments [73].

Wines with high tannin levels are reported to have a longer aging ability [48]. If all the conditions of wine storage are properly controlled, the tannins in red wine can evolve in a positive direction to reduce astringency [74]

Norisoprenoids are key contributors to wine aroma development [75]. Among them, 1,1,6-trimethyl-1,2-dihydronaphthalene (TDN) is commonly associated with undesirable kerosene or cooked meat notes and is considered an indicator of premature oxidation, although at low concentrations it may impart caramel-like aromas and is characteristic of Riesling wines [76,77]. Other norisoprenoids, such as β-damascenone and β-ionone, increase during bottle aging as a result of oxidative degradation of grape carotenoids. These compounds contribute floral and fruity notes and can enhance the perception of other aromatic constituents [78].

Terpene alcohols (terpenols) derived from monoterpenes play an important role in aroma evolution during bottle aging. The main terpenols in wine include geraniol, linalool, and α-terpineol [79]. Their concentrations change over time due to acid-catalyzed hydrolysis and redox reactions, often showing an initial increase followed by a decline during prolonged storage, a pattern observed across various wine styles [20,80,81].

Most aromatic thiols are formed during alcoholic fermentation, and bottle aging primarily serves to preserve them from degradation [82]. Nevertheless, certain thiols may develop during aging, including benzenemethanethiol and 2-furanmethanethiol, both characterized by extremely low perception thresholds and roasted or mineral-like aromas [26]. Benzaldehyde has been proposed as a precursor of benzenemethanethiol, while the formation of 2-furanmethanethiol appears to be associated with a decrease in furfural content during aging [83,84]. Another relevant thiol, 2-methyl-3-furanthiol, contributes “toasty” and cooked meat notes to the wine aroma [83].

### 2.8. Negative Change in Bottling Aging

#### Excessive Oxidation

Excessive oxidation, caused either by high oxygen transmission rates during storage or by poorly controlled storage conditions, can have detrimental effects on wine quality. The most evident consequences include alterations in color, the development of oxidative off-odors, and the loss of varietal aromatic characteristics [53]. Color deterioration is typically expressed as wine “browning,” a defect particularly noticeable in white wines, where it leads to reduced clarity and unattractive hues; notable exceptions are white Port and Sherry wines, for which such coloration is considered typical [66].

A major factor contributing to browning is the accumulation of xanthylium cations, which impart yellow tones and, upon further oxidation, can give rise to polymeric brown pigments [85]. Although the precise chemical structures, origins, and formation pathways of these non-enzymatically generated brown pigments are not yet fully understood [86], their presence has been associated with declining levels of precursor compounds such as flavonoids and anthocyanins, along with increasing concentrations of xanthylium salts [26].

From an aromatic standpoint, highly oxidized wines often show intensified oxidation of ethanol, leading to elevated levels of acetaldehyde and acetic acid, which mask desirable aromas and impart characteristic oxidative odors [87,88]. Acetic acid can originate both from microbial metabolism during fermentation and from subsequent oxidation of acetaldehyde during bottle storage [25]. Acetaldehyde itself, continuously generated from ethanol oxidation, plays a central role in oxidative wine evolution and may eventually dominate the aroma profile, in addition to participating in several chemical reactions such as pyranoanthocyanin formation.

Furthermore, oxidative imbalance promotes the formation of undesirable aldehydes through Strecker degradation of amino acids mediated by quinones [36,37,65]. Methional is the most characteristic aldehyde in oxidized wines and is associated with “boiled potato” aromas [89]. Phenylacetaldehyde, derived from phenylalanine degradation, represents another key oxidation marker: while it may contribute sweet and floral notes at low concentrations, higher levels, typical of severely oxidized wines, produce green or moss-like off-odors [70]. In addition, some esters formed during bottle aging, particularly acetates resulting from reactions involving aldehydes, can further contribute to undesirable aromatic notes, with ethyl acetate being the most abundant ester in wine.

### 2.9. Alternative Aging Aproaches

In view of the oxidative mechanisms described above, the evolution of wine in the bottle does not depend exclusively on its initial chemical composition, but the composition is influenced by a series of physical factors linked to storage conditions. Parameters such as the type of closure, storage time, temperature and exposure to light play a decisive role in modulating the aging processes, influencing both positive aromatic evolution and the risk of oxidative degradation.

In ancient times, before the widespread use of glass bottles and cork closures, wines were rarely stored for long periods. The containers commonly used, such as clay amphorae, offered limited protection against oxygen ingress, making prolonged storage impractical [90]. Oxidative phenomena were further aggravated by a lack of knowledge about the protective role of sulphur dioxide. As a result, wine was generally consumed within a year of harvest, and new wine was often preferred despite its sensory instability [20].

The production techniques used were more akin to oxidative or sweet wines, comparable to today’s Sherry or Madeira, rather than modern table wines. A fundamental change came in the 18th century with the widespread introduction of more resistant glass bottles and the use of Quercus suber cork as a closure system, which significantly reduced oxygen transfer and made it possible to store wine in bottles for long periods of time [20].

With centuries of experience gained from this packaging system, consumers and critics have developed precise expectations about how wine evolves in the bottle [91]. These expectations have been largely shaped by the traditional conditions of European cellars, where underground storage ensured cool, stable temperatures and minimal seasonal variations. Regions such as Bordeaux, with an average cellar temperature of around 13 °C in the 20th century, became a benchmark for conditions considered ideal for wine aging. In addition, the high humidity of underground cellars helped to preserve the integrity of the closures, further reinforcing this model of storage [20].

In light of this historical context, a fundamental question arises: is it possible to develop alternative aging strategies that can reproduce the effects of traditional aging while reducing the time required? Since wine aging is governed by slow physical–chemical processes, and many iconic wines require decades before being marketed, accelerated aging has been the subject of numerous studies.

#### 2.9.1. High Storage Temperatures and Accelerated Aging

The speed of many chemical reactions involved in wine aging increases with rising temperatures, as seen above. Consequently, lower storage temperatures are generally more effective in preserving the sensory characteristics of young wines [20]. Chemical reactions in wine are characterized by different activation energies, which can be described by the Arrhenius equation [24]. Temperature also affects equilibrium constants, including those related to acid dissociation, resulting in lower pKa values and an increase in pH-dependent reactions at higher temperatures. Since different reactions respond unevenly to temperature changes, attempts to accelerate aging by heating often lead to different reaction pathways rather than simply accelerating natural aging. This heterogeneity represents a significant limitation for the application of strategies based on increasing temperature.

A study conducted by Cejudo-Bastante et al. provides an illustrative example of these effects. A Chardonnay wine aged for one year at 18 °C was compared with the same wine stored for seven days at 50 °C. Although both showed changes attributable to aging, the chemical results differed substantially. Ethyl hexanoate increased similarly in both cases, while hexyl acetate decreased more markedly at lower temperatures. Diethyl succinate, often considered a marker of aging, increased significantly more at 18 °C than with heat treatment. These results demonstrate that raising the temperature does not accurately reproduce the chemistry of traditional aging. As a result, the systematic use of heat to accelerate aging is not widespread, except for specific wine styles such as Madeira [92].

#### 2.9.2. Increase in Oxygen

Although oxidation can contribute positively to sensory evolution, it also represents a limiting factor for shelf life [24,25]. In addition to natural exposure to oxygen during aging, micro-oxygenation was introduced in the 1990s as a technique to accelerate aging in barrels or tanks, becoming an established tool in modern oenology [93].

Micro-oxygenation consists of the controlled and continuous introduction of very small quantities of oxygen into the wine, generally expressed in milligrams of oxygen per liter per month, using devices that allow for extremely precise dosing. This technique makes it possible to reproduce, in a short time, the conditions of slow oxygenation that occur naturally through the porosity of the wood of the barrels, while reducing the risks associated with uncontrolled oxidative exposure. From a chemical point of view, micro-oxygenation mainly influences the redox reactions of phenolic compounds [54,94]. In particular, it promotes the polymerization of tannins and their interaction with anthocyanins, contributing to the stabilization of color in red wines and the reduction in astringency. The oxygen introduced is rapidly consumed in reactions catalyzed by transition metals, such as iron and copper, limiting the accumulation of dissolved oxygen and preventing unwanted oxidative phenomena [93].

The sensory effects of micro-oxygenation include a softening of the tannic structure, greater roundness on the palate and an overall better balance of the wine. In addition, this technique can reduce the perception of vegetal or reductive notes and promote faster aromatic evolution. However, the effectiveness of micro-oxygenation depends on numerous factors, including the phenolic composition of the wine, temperature, sulphur dioxide content and the timing of the treatment. Inadequate dosage or insufficient control can lead to premature oxidation and a loss of aromatic freshness [95].

#### 2.9.3. Mechanical Movement and Ultrasound

The use of mechanical energy, in the form of agitation, mixing or vibration, has historically been explored as a possible means of accelerating wine aging [20].

However, recent studies indicate that bottle vibration has negligible effects on sulphur dioxide levels, dissolved oxygen or volatile compound content, although some impact on ester balance has been observed [96]. From a theoretical point of view, the diffusion coefficients of small molecules in wine are high enough to make it unlikely that mass transport will limit aging reactions. Therefore, the effects observed could be attributed more to accidental heating of the samples than to a real improvement in molecular mixing [97].

Ultrasonic treatment has also been studied as a possible method of accelerating aging. Although ultrasound can promote certain chemical reactions and the release of polysaccharides from yeast lees, there is limited evidence to support a real extension of shelf life or effective simulation of aging [98,99].

#### 2.9.4. Radiation

Electromagnetic radiation can trigger chemical reactions and potentially affect the aging of wine. The effects depend heavily on the wavelength used. Although ultraviolet and visible radiation are widely used in synthetic chemistry to target specific functional groups, their application to wine aging has shown unfavorable results. Exposure to light, particularly UV rays, is known to induce sensory defects in white wines and accelerate quality decay [100,101].

Ionizing radiation, such as X-rays and gamma rays, has also been evaluated as a possible tool for accelerated aging. Although effective for sterilization in the food industry, the results obtained in wine have been mixed. Some studies have shown a reduction in redox potential and an increase in aldehydes, accompanied by color loss and deterioration in sensory quality at high doses. Despite the absence of radioactive residues in the treated samples, limited sensory improvements and consumer concerns have hampered the development of this technology in oenology [20,102].

In addition to physical and chemical strategies, recent developments in digital oenology are opening new perspectives for monitoring and managing wine bottle aging. IoT-based sensing systems allow real-time tracking of key storage parameters such as temperature, pressure, light exposure and, potentially, oxygen dynamics, enabling more precise control of aging conditions [103]. In parallel, machine learning approaches are increasingly explored to relate chemical fingerprints, environmental variables and sensory descriptors, with the aim of predicting wine evolution during bottle storage. These data-driven tools may support the optimization of aging strategies, including emerging approaches such as underwater storage, by integrating environmental monitoring with predictive modeling of phenolic, aromatic and color changes. Such developments are consistent with the concept of precision winemaking, in which storage conditions are actively monitored and managed to guide wine evolution.

In this context, the aim of the present review is to provide a critical and integrated overview of the main mechanisms governing wine bottle aging and of the emerging strategies proposed to modulate wine evolution during storage. Particular attention is given not only to oxidative chemistry and storage-related factors, but also to the current limits of accelerated aging techniques, the still preliminary evidence on underwater aging, and the broader implications of sustainability, climate change and digital monitoring for precision wine aging. By combining these perspectives, this review seeks to move beyond a purely descriptive summary and to identify the main challenges and research priorities for future developments in wine bottle refinement.

#### 2.9.5. Underwater Aging

Interest in underwater aging of wine stems from historical accounts linked to maritime transport and the accidental recovery of ancient bottles from the seabed. Underwater wine aging has enjoyed increasing success in recent years, because it represents an innovation that can boost the range of products on offer. From a physical–chemical point of view, underwater aging combines several storage conditions traditionally considered favorable for wine aging. Submerged environments are characterized by low and highly stable temperatures, darkness and limited exposure to external oxygen sources. In addition, hydrostatic pressure and continuous micro-movements induced by water currents introduce physical variables that are absent in traditional cellar storage. The simultaneous action of these factors can influence gas solubility, mass transfer processes and reaction kinetics during bottle aging, potentially leading to distinct aging trajectories.

Despite growing commercial interest, systematic scientific evidence supporting underwater aging remains scarce. As a result, underwater aging is currently considered an experimental or niche practice rather than a validated aging strategy.

A few studies have addressed this issue.

Recent work by Birkić et al. (2024) investigated the maturation of wines aged in underwater springs compared with conventional cellar storage [104]. The authors reported no significant differences in total phenolic content, flavonoid levels or overall antioxidant capacity between the two aging conditions. However, LC–MS/MS analysis revealed significantly higher concentrations of specific flavonoids, particularly naringenin and myricetin, in underwater-aged wines. These compounds are known for their strong antioxidant activity, which suggests that the lower temperature and reduced exposure to oxygen in submerged environments may help to preserve certain phenolic compounds rather than causing significant changes in their composition. This evidence supports the hypothesis that underwater aging may modulate wine evolution through selective stabilization of antioxidant molecules [104].

A study conducted by Balivo et al. assessed the impact of underwater aging on the chemical, phenolic and aromatic composition of a wine-based liqueur (Elixir Falernum), comparing bottles aged for 12 months at a depth of 13 m at the following coordinates (latitude 41°07′21.9″, longitude 13°50′48.541″) with a control aged in the cellar [105].

The bottles, from the same production batch, were sealed and kept in a horizontal position; at the end of the aging period, the samples were analyzed using an electronic nose, basic chemical analyses (pH, alcohol content, acidity), phenolic determinations (total polyphenols, anthocyanins), CIELab colorimetric parameters and analysis of volatile compounds using SPME-GC/MS.

**Figure 6:**
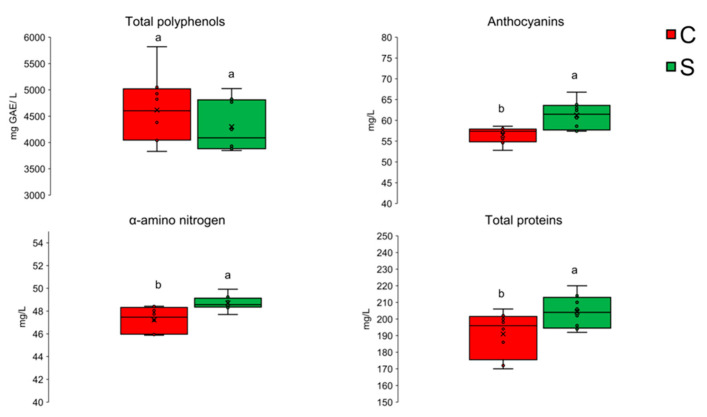
Concentration of phenols, anthocyanins, and α-amino nitrogen proteins in samples aged at sea (S) and in the cellar (C). Reprinted from reference [105]. Data are expressed as mean ± SD; the letters (a, b) indicate significant differences (*p* < 0.05) after the analysis of variance (ANOVA).

The results showed no significant differences between the two treatments in terms of pH, alcohol content, and total and volatile acidity (Table 2). However, the samples aged at sea showed a significantly higher anthocyanin content, associated with a lower yellow index (b), suggesting less oxidative degradation of phenolic pigments in an underwater environment. At the same time, higher levels of free α-amino nitrogen and total proteins were observed, indicating a slowdown in oxidative processes (Figure 6).

From an aromatic point of view, the analysis of volatile compounds revealed a significant increase in samples aged at sea in furanones and pyranones such as 5-hydroxymethylfurfural, furaneol and 3,5-dihydroxy-6-methyl-2,3-dihydro-4H-pyran-4-one, compounds associated with fruity, toasted and caramelized notes. Analysis using an electronic nose confirmed these differences, allowing the samples to be correctly classified with 96% accuracy. The authors attribute these effects to the unique conditions of the underwater environment, characterized by reduced oxygen availability, more stable temperatures, vibrations and blue-green light, which seem to slow down oxidative evolution and modulate the aromatic development of the product [105].

A recent study conducted by Mercanti et al. allowed two types of wine (Sangiovese and Merlot) to age in the Mediterranean Sea at a depth of 25 m in the Gulf of Follonica [106].

The bottles were packaged according to the scheme shown in Table 3.

**Figure 7:**
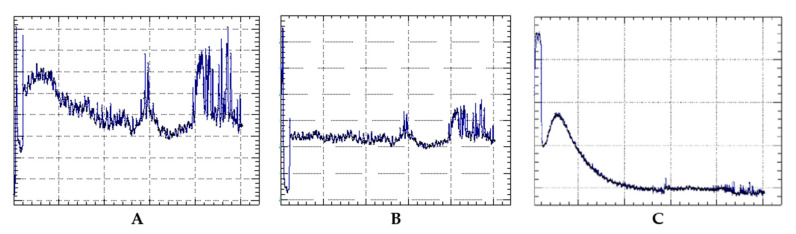
Trend of the pressure (**A**) and temperature (**B**) variables and evolution of the volume (**C**) of the headspace of the bottles during the entire aging period, as a function of time. Reprinted from reference [106].

Tailor-made sensors have been developed to monitor physical parameters inside the bottles, such as temperature and pressure, in order to verify the possible correlation between changes in these parameters and changes in compounds during the underwater aging period (Figure 7).

**Figure 8:**
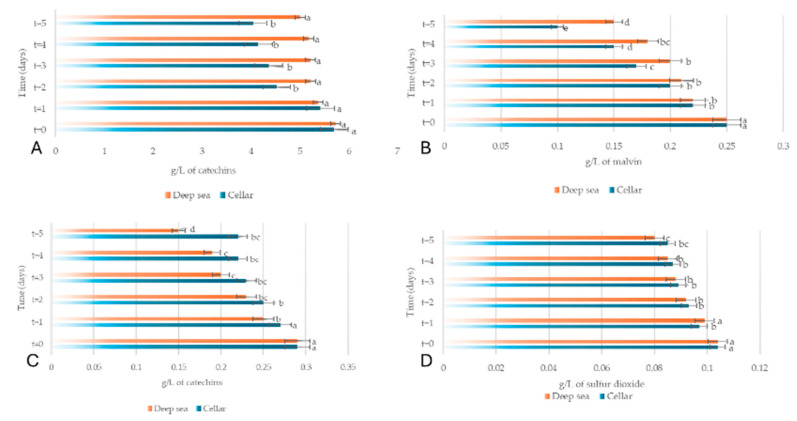
Phenolic content (g/L) (**A**), anthocyanin content (mg/L) (**B**), non-flavonoid content (g/L) (**C**) and sulphur dioxide content (mg/L) (**D**) of Merlot wine. Wine aged in the cellar is shown in blue; wine aged at sea is shown in orange. The letters (a–e) indicate significant differences (*p* < 0.05) over time, after ANOVA analysis of variance. Reprinted from reference [106].

**Figure 9:**
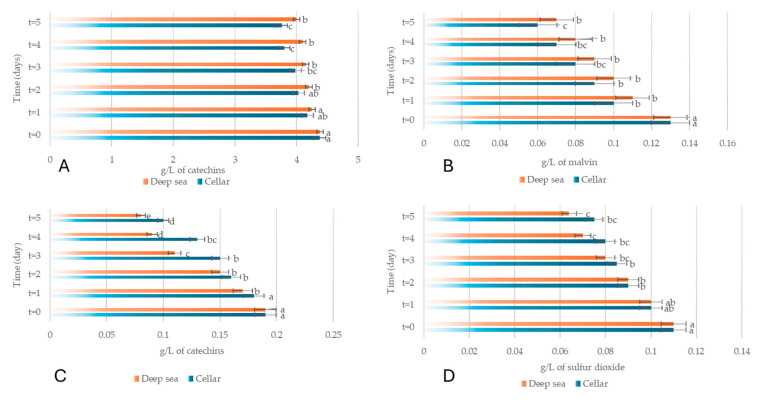
Phenolic content (g/L) (**A**), anthocyanin content (mg/L) (**B**), non-flavonoid content (g/L) (**C**) and sulphur dioxide content (mg/L) (**D**) of Merlot wine. Wine aged in the cellar is shown in blue; wine aged at sea is shown in orange. The letters (a–e) indicate significant differences (*p* < 0.05) over time, after ANOVA analysis of variance. Reprinted from reference [106].

Chemical analyses showed that, both in the cellar and underwater, Merlot and Sangiovese underwent a decrease in total phenols, total anthocyanins, non-flavonoid compounds and total sulphur dioxide, in line with oxidation and other reactions typical of wine aging; however, underwater aging seemed to better preserve phenolic and anthocyanin content than cellar storage (Figure 8 and Figure 9). Overall, the data showed that underwater aging did not significantly alter the basic composition of the wines compared to traditional cellar aging, but it did subtly influence the evolution of color and structural compounds, highlighting the complex interaction between oxygen and storage conditions in wine quality during aging.

A further study by Maioli et al. demonstrated lower astringency and a greater aroma of red fruits and balsamic notes in wine aged at sea [107].

The bottles, from the same production batch and sealed with natural or technical corks, were submerged for six months at a depth of 52 m in the Ligurian Sea, in conditions of darkness, stable temperature, high pressure (6.3 bar) and slight current movement.

At the end of the six months (t6), some of the underwater bottles and the control bottles were analyzed; subsequently, both were subjected to a further six months of aging in the cellar (t12) to assess their evolution over time. The analyses included: standard chemical parameters (pH, acidity, alcohol, SO_2_), elemental profile (ICP), volatile profile (SPME-GC/MS and GC-FID), phenolic composition (HPLC-DAD), CIELab colorimetric parameters and discriminating sensory tests (triangle test) [107].

The results showed that underwater aging did not alter the standard chemical parameters of the wines (alcohol content, pH, acidity) (Table 4), confirming the integrity of the bottles and the absence of seawater contamination. The main differences between wines aged underwater and in the cellar concerned the phenolic and colorimetric profile, while the volatile fraction was less affected by the treatment.

After 6 months of aging (t6), the underwater wines, particularly rosé and red, showed lighter colors, higher L* values and higher monomeric anthocyanin content, but lower levels of polymeric pigments, suggesting a slower oxidative evolution than the cellar wines. In red wines, the color difference between the two treatments was visually perceptible (ΔE > 3), while in white and rosé wines it was more subtle (Table 5).

After a further 6 months in the cellar (t12), the differences between underwater and control wines tended to decrease, indicating that underwater aging mainly affects the initial stages of wine evolution, slowing down oxidative and phenolic polymerization processes (Table 5). Sensory discrimination tests confirmed that wines aged underwater and in the cellar were perceived as different, with the effects depending on the type of wine and the time of evaluation.

Overall, the study suggests that underwater aging represents an alternative method of oxygen management, capable of modulating the phenolic and chromatic evolution of wine in a different way compared to traditional aging, with potential implications for controlling oxidative phenomena during maturation.

Beyond the oenological aspects, underwater aging should also be considered from a sustainability perspective. In principle, submerged environments can provide naturally stable temperatures and protection from light, potentially reducing the need for energy-intensive temperature control, typically required in temperature-controlled storage facilities. However, any potential environmental benefits must be assessed in light of the logistical requirements associated with sea-based installation, recovery operations, packaging requirements and site-specific maintenance. For this reason, future studies should integrate chemical and sensory assessments with life-cycle analysis (LCA) approaches in order to compare underwater aging with conventional cellar storage under controlled conditions.

Future research should focus on well-controlled comparative studies between underwater aging and conventional aging in cellars, with particular attention to oxygen dynamics, redox potential, volatile composition and sensory evolution over time. Only through further investigation will it be possible to assess whether underwater aging represents a real alternative or remains primarily a niche and experimental practice.

### 2.10. Climate Change and Bottle Aging Perspective

Alongside the growing interest in alternative aging strategies, it is also necessary to consider the impact of climate change on the composition of grapes and wine. Warmer growing conditions are often associated with earlier maturation, greater sugar accumulation, increased ethanol content, lower total acidity and, in many cases, higher pH values [108]. These compositional changes are particularly relevant to bottle aging, as they can influence the redox balance, the kinetics of oxygen consumption, color stability and the overall sensory evolution of wines during storage [81,109]. Higher pH conditions can reduce microbial and chemical stability, whilst higher alcohol levels can alter the extraction, reactivity and perception of phenolic and aromatic compounds. Consequently, it is anticipated that climate-driven changes in wine composition will influence not only winemaking decisions but also the aging potential and storage strategies required for white, rosé and red wines [110,111,112,113].

## 3. Conclusions

The aging of wine in the bottle is a complex process, governed by a network of chemical and physical–chemical reactions that are strongly influenced by storage conditions. As highlighted in this study, factors such as the type of closure, storage time, temperature and exposure to light play a decisive role in modulating the aromatic, chromatic and structural evolution of wine, affecting both the positive aspects of aging and the risk of oxidative degradation.

Oxidative mechanisms, mediated by the interaction between oxygen, phenolic compounds, transition metals and sulphur dioxide, are central to the evolution of wine in the bottle. Inadequate control of these parameters can lead to excessive oxidation, loss of varietal aromas and undesirable color changes, while optimal management allows for the development of sensory complexity and stability over time.

Over the years, numerous alternative approaches have been proposed to accelerate or modulate wine aging, including increasing temperature, micro-oxygenation, the application of mechanical movements, ultrasound and various forms of radiation. However, most of these techniques have proven incapable of faithfully reproducing the chemical pathways of traditional aging, often generating different aromatic profiles or undesirable side effects. As a result, their use remains limited to experimental contexts or specific wine styles.

In this context, underwater aging emerges as one of the most innovative and fascinating techniques. Based on environmental conditions characterized by low temperature, absence of light, high pressure and considerable thermal stability, this approach is conceptually similar to the principles of traditional aging in cellars, while introducing unique physical variables. The few studies available indicate that aging in a marine environment does not lead to a deterioration in wine quality and may, in some cases, promote distinctive sensory profiles, with greater tannic softness and specific aromatic notes.

Future studies should combine controlled comparative aging trials, monitoring of oxygen dynamics and redox potential, targeted and untargeted phenolic and volatile profiling, sensory analysis, sustainability assessment, and digital monitoring tools in order to comprehensively evaluate wine evolution under conventional and emerging aging conditions.

## Figures and Tables

**Table 1:** Classification of the main types of wine found around the world. Adapted from reference [20].

Description	Color	Residual Sugar (g/L)	Alcohol (% *v*/*v*)	Examples
**White** **wines**				
Still—Dry	Pale straw to gold	<9	8 to 14.5	Riesling, Chardonnay, Semillon, Sauvignon Blanc, Colombard, Grüner Veltliner, Trebbiano, Chenin Blanc
Still—Sweet	Light yellow to gold	9 to 30 (semi-sweet) 30 to 200 (sweet)	8 to 14.5	Riesling, Gewürztraminer, Semillon, Ice wines, Sauternes, Tokay
Sparkling—Dry to semi-sweet	Pale straw to amber (pink to light red for rosé styles)	0 to 50	10 to 12.5	Champagne, Chardonnay/Pinot Noir/Pinot Meunier, Riesling, Sauvignon Blanc, Cava, Prosecco
Fortified—Dry	Pale straw to amber	0 to 30	15 to 20.5	Fino and Amontillado Sherries
Fortified—Sweet	Pale straw to amber	100 to 300	15 to 20.5	Oloroso and Pedro Ximénez Sherries, Topaque
**Rosé wines**				
Still—Dry to semi-sweet	Pale pink to salmon	<9 (dry) 9 to 30 (semi-sweet)	8 to 14	Rosé de Provence, Grenache rosé, Pinot Noir rosé, Tempranillo rosé, White Zinfandel
Sparkling—Dry to semi-sweet	Pale pink to light red	0 to 50	10 to 12.5	Rosé Champagne, Rosé Cava, Rosé Prosecco
**Red wines**				
Still—Dry	Dark red to red/brown	<7.5	8 to 14.5	Grenache, Merlot, Cabernet Sauvignon, Tempranillo, Zinfandel, Sangiovese, Malbec, Pinot Noir, Shiraz
Sparkling—Semi-sweet	Red to red/brown	7.5 to 30	10 to 12.5	Sparkling Pinot Noir, Shiraz, Cabernet Sauvignon
Fortified—Sweet	Red/gold to deep brown	100 to 300	18 to 22	Ruby, Tawny and Vintage Ports, Brown Muscat

**Table 2:** Analysis of ethanol (%*v*/*v*), titratable acidity (g/L), volatile acidity (g/L), pH and CIElab coordinates (a, b, L). Adapted from reference [105].

Parameter	S (Underwater Aging)	C (Cellar Aging)
Ethanol (% *v*/*v*)	26.27 ± 0.12 ^a^	26.10 ± 0.10 ^a^
Titratable acidity (g/L)	3.23 ± 0.14 ^a^	3.21 ± 0.15 ^a^
Volatile acidity (g/L)	0.21 ± 0.01 ^a^	0.215 ± 0.01 ^a^
pH	3.66 ± 0.01 ^a^	3.66 ± 0.02 ^a^
L*	26.16 ± 0.15 ^a^	25.57 ± 0.74 ^a^
a*	−2.61 ± 0.51 ^a^	−3.51 ± 1.52 ^a^
b*	4.59 ± 0.59 ^b^	5.97 ± 1.19 ^a^

Data are expressed as mean ± SD; in the same column, the letters (a, b) indicate significant differences (*p* < 0.05) after the analysis of variance (ANOVA).

**Table 3:** Layout for packaging bottles before placing them in the sea. Reprinted from reference [106].

Wine Type	Cork Type	Capsule Type	Wrapping	Bottle Orientation	Storage Location
Sangiovese 2019	Monoblock cork	Shellac capsule	Wrapped	Held upright	Under sea
Merlot 2020	Monoblock cork	Shellac capsule	Wrapped	Held upright	Under sea
Sangiovese 2019	Monoblock cork	Shellac capsule	Wrapped	Held upright	Cellar
Merlot 2020	Monoblock cork	Shellac capsule	Wrapped	Held upright	Cellar

**Table 4:** Comparison of alcohol content (%*v*/*v*), residual sugars (g/L), titratable acidity (g/L), pH, volatile acidity (g/L), and sulphur dioxide (mg/L) in wines aged at sea and in the cellar. Adapted from reference [107].

Wine	Type	Aging	Alcohol (% *v*/*v*)	Residual Sugars (g/L)	Titratable Acidity (g/L)	pH	Volatile Acidity (g/L)	Free SO_2_ (mg/L)	Total SO_2_ (mg/L)
BT	White	Cellar	13.51 ± 0.01	4.43 ± 0.05	6.78 ± 0.03	3.20 ± 0.01	0.15 ± 0.01	16 ± 3	52 ± 1
BM	White	Underwater	13.51 ± 0.04	4.38 ± 0.08	6.78 ± 0.02	3.20 ± 0.01	0.15 ± 0.01	16 ± 3	62 ± 1
PT	Rosé	Cellar	13.32 ± 0.01	n.d.	5.82 ± 0.01	3.29 ± 0.01	0.34 ± 0.01	12 ± 1	54 ± 1
PM	Rosé	Underwater	13.29 ± 0.07	n.d.	5.78 ± 0.03	3.29 ± 0.01	0.34 ± 0.02	12 ± 1	56 ± 1
RT	Red	Cellar	13.92 ± 0.01	0.54 ± 0.01	5.85 ± 0.02	3.36 ± 0.01	0.46 ± 0.01	14 ± 1	49 ± 1
RM	Red	Underwater	13.86 ± 0.01	0.54 ± 0.05	5.90 ± 0.01	3.33 ± 0.01	0.45 ± 0.01	14 ± 1	37 ± 1

**Table 5:** Comparison of anthocyanin content and CIElab coordinates for red and rosé wines aged at sea and in the cellar. Adapted from reference [107].

Wine	Time	Aging	Anthocyanins (mg/L)	L*	a*	b*	ΔE
**Rosé**	t6	Cellar (PT)	Malv-3-O-glc: 3.01 ± 0.01	90.11 ± 0.23	7.98 ± 0.19	18.78 ± 0.54	–
	t6	Underwater (PM)	Malv-3-O-glc: 4.34 ± 0.58	91.55 ± 0.07	7.43 ± 0.12	16.80 ± 0.50	2.53
	t12	Cellar (PT)	Malv-3-O-glc: 2.91 ± 0.06Acylated anthocyanin #4: 4.32 ± 0.03	90.98 ± 0.06	7.28 ± 0.04	n.r.	–
	t12	Underwater (PM)	Malv-3-O-glc: 3.52 ± 0.27Acylated anthocyanin #4: 3.45 ± 0.09	91.36 ± 0.12	7.01 ± 0.10	n.r.	0.63
**Red**	t6	Cellar (RT)	n.r.	22.18 ± 0.78	52.85 ± 0.85	37.65 ± 0.85	–
	t6	Underwater (RM)	n.r.	18.56 ± 0.39	49.35 ± 0.51	31.76 ± 0.66	7.74
	t12	Cellar (RT)	Total monomeric anthocyanins: 98.80 ± 1.61	n.r.	n.r.	n.r.	–
	t12	Underwater (RM)	Total monomeric anthocyanins: 114.02 ± 5.87Delph-3-O-glc: 4.97 ± 0.64	n.r.	n.r.	n.r.	1.12

## Data Availability

The original contributions presented in the study are included in the article. Further inquiries can be directed to the corresponding author.

## References

[1] Sáenz-Navajas M.-P., Jeffery D.W. (2021). Perspectives on Wines of Provenance: Sensory Typicality, Quality, and Authenticity. ACS Food Sci. Technol..

[2] European Parliament and Council of the European Union (2013). Regulation (EU) No 1308/2013 of the European Parliament and of the Council of 17 December 2013 Establishing a Common Organisation of the Markets in Agricultural Products and Repealing Council Regulations (EEC) No 922/72, (EEC) No 234/79, (EC) No 1037/2001. Off. J. Eur. Communities.

[3] World Flora Online—WFO. https://www.worldfloraonline.org/.

[4] http://www.worldfloraonline.org/taxon/wfo-7000000318#children.

[5] Keller M. (2020). Taxonomy and Anatomy. The Science of Grapevines.

[6] Fortes A.M., Pais M.S. (2015). Grape (Vitis Species). Nutritional Composition of Fruit Cultivars.

[7] Venkitasamy C., Zhao L., Zhang R., Pan Z. (2019). Grapes. Integrated Processing Technologies for Food and Agricultural By-Products.

[8] Grainger K., Tattersall H. (2005). Wine Production: Vine to Bottle.

[9] Van Leeuwen C., Barbe J.-C., Garbay J., Gowdy M., Lytra G., Plantevin M., Pons A., Thibon C., Marchand S. (2023). Aromatic Ripeness May Be the Type of Maturity That Impacts Red Wine Typicity the Most. Part I: The Aromas Involved in Aromatic Ripeness: Sourced from the Research Article: “Aromatic Maturity Is a Cornerstone of Terroir Expression in Red Wine” (OENO One, 2022). Original Language of the Article: English. IVES Tech. Rev. Vine Wine.

[10] Rajha H., Darra N., Kantar S., Hobaika Z., Louka N., Maroun R. (2017). A Comparative Study of the Phenolic and Technological Maturities of Red Grapes Grown in Lebanon. Antioxidants.

[11] López R., Portu J., González-Arenzana L., Garijo P., Gutiérrez A.R., Santamaría P. (2021). Ethephon Foliar Application: Impact on the Phenolic and Technological Tempranillo Grapes Maturity. J. Food Sci..

[12] Polat B. (2021). Reduction of Some Insecticide Residues from Grapes with Washing Treatments. Turk. J. Entomol..

[13] Maggu M., Winz R., Kilmartin P.A., Trought M.C.T., Nicolau L. (2007). Effect of Skin Contact and Pressure on the Composition of Sauvignon Blanc Must. J. Agric. Food Chem..

[14] Sicheri G. (2015). Enologia. Con Elementi Di Chimica Viticolo-Enologica.

[15] Casassa L.F., Soto-Hernández M., Palma-Tenango M., Garcia-Mateos M.D.R. (2017). Flavonoid Phenolics in Red Winemaking. Phenolic Compounds—Natural Sources, Importance and Applications.

[16] Canals R., Llaudy M.C., Valls J., Canals J.M., Zamora F. (2005). Influence of Ethanol Concentration on the Extraction of Color and Phenolic Compounds from the Skin and Seeds of Tempranillo Grapes at Different Stages of Ripening. J. Agric. Food Chem..

[17] Suriano S., Basile T., Tarricone L., Di Gennaro D., Tamborra P. (2015). Effects of Skin Maceration Time on the Phenolic and Sensory Characteristics of Bombino Nero Rosé Wines. Ital. J. Agron..

[18] Granès G. (2008). La Fermentation Alcoolique. Le Vin Rosé.

[19] Flamini R. (2008). Hyphenated Techniques in Grape and Wine Chemistry.

[20] Waterhouse A.L., Sacks G.L., Jeffery D.W. (2024). Understanding Wine Chemistry.

[21] Gutiérrez-Escobar R., Aliaño-González M.J., Cantos-Villar E. (2021). Wine Polyphenol Content and Its Influence on Wine Quality and Properties: A Review. Molecules.

[22] Maza M.A., Pereira C., Martínez J.M., Camargo A., Álvarez I., Raso J. (2020). PEF Treatments of High Specific Energy Permit the Reduction of Maceration Time during Vinification of Caladoc and Grenache Grapes. Innov. Food Sci. Emerg. Technol..

[23] De Lima P.H.N., Rodrigues C.F., De Arruda J.H.B., Santos A.M.P., De Andrade Lima L.L. (2023). Physicochemical Changes in Red Wines from the São Francisco Valley (Brazil) after Aged in a Bottle. Int. J. Food Sci. Technol..

[24] Mercanti N., Macaluso M., Pieracci Y., Brazzarola F., Palla F., Verdini P.G., Zinnai A. (2024). Enhancing Wine Shelf-Life: Insights into Factors Influencing Oxidation and Preservation. Heliyon.

[25] Karbowiak T., Gougeon R.D., Alinc J.-B., Brachais L., Debeaufort F., Voilley A., Chassagne D. (2009). Wine Oxidation and the Role of Cork. Crit. Rev. Food Sci. Nutr..

[26] Echave J., Barral M., Fraga-Corral M., Prieto M.A., Simal-Gandara J. (2021). Bottle Aging and Storage of Wines: A Review. Molecules.

[27] Danilewicz J.C. (2003). Review of Reaction Mechanisms of Oxygen and Proposed Intermediate Reduction Products in Wine: Central Role of Iron and Copper. Am. J. Enol. Vitic..

[28] Danilewicz J.C., Seccombe J.T., Whelan J. (2008). Mechanism of Interaction of Polyphenols, Oxygen, and Sulfur Dioxide in Model Wine and Wine. Am. J. Enol. Vitic..

[29] Perez-Benito J.F. (2004). Iron(III)−Hydrogen Peroxide Reaction: Kinetic Evidence of a Hydroxyl-Mediated Chain Mechanism. J. Phys. Chem. A.

[30] Ribéreau-Gayon P., Glories Y., Maujean A., Dubourdieu D. (2004). Ribéreau-Gayon les Phénols Volatils Responsables des Certaines Déviations Olfactives de Type <Phénolé> des Vins.

[31] Vangijzegem T., Lecomte V., Ternad I., Van Leuven L., Muller R.N., Stanicki D., Laurent S. (2023). Superparamagnetic Iron Oxide Nanoparticles (SPION): From Fundamentals to State-of-the-Art Innovative Applications for Cancer Therapy. Pharmaceutics.

[32] Ugliano M. (2013). Oxygen Contribution to Wine Aroma Evolution during Bottle Aging. J. Agric. Food Chem..

[33] Kreitman G.Y., Laurie V.F., Elias R.J. (2013). Investigation of Ethyl Radical Quenching by Phenolics and Thiols in Model Wine. J. Agric. Food Chem..

[34] Giacosa S., Río Segade S., Cagnasso E., Caudana A., Rolle L., Gerbi V. (2019). SO_2_ in Wines. Red Wine Technology.

[35] Sioumis N., Kallithraka S., Tsoutsouras E., Makris D.P., Kefalas P. (2005). Browning Development in White Wines: Dependence on Compositional Parameters and Impact on Antioxidant Characteristics. Eur. Food Res. Technol..

[36] Ribéreau-Gayon J., Peynaud E. (1957). Trattato di Enologia.

[37] Minussi R.C., Rossi M., Bologna L., Cordi L., Rotilio D., Pastore G.M., Durán N. (2003). Phenolic Compounds and Total Antioxidant Potential of Commercial Wines. Food Chem..

[38] Wirth J., Morel-Salmi C., Souquet J.M., Dieval J.B., Aagaard O., Vidal S., Fulcrand H., Cheynier V. (2010). The Impact of Oxygen Exposure before and after Bottling on the Polyphenolic Composition of Red Wines. Food Chem..

[39] Cheynier V., Masson G., Rigaud J., Moutounet M. (1993). Estimation of Must Oxidation During Pressing in Champagne. Am. J. Enol. Vitic..

[40] Oliveira C.M., Barros A.S., Silva Ferreira A.C., Silva A.M.S. (2015). Influence of the Temperature and Oxygen Exposure in Red Port Wine: A Kinetic Approach. Food Res. Int..

[41] Bonamente E., Scrucca F., Rinaldi S., Merico M.C., Asdrubali F., Lamastra L. (2016). Environmental Impact of an Italian Wine Bottle: Carbon and Water Footprint Assessment. Sci. Total Environ..

[42] Guaita M., Petrozziello M., Motta S., Bonello F., Cravero M.C., Marulli C., Bosso A. (2013). Effect of the Closure Type on the Evolution of the Physical-Chemical and Sensory Characteristics of a Montepulciano d’Abruzzo *Rosé* Wine. J. Food Sci..

[43] Gambuti A., Rinaldi A., Ugliano M., Moio L. (2013). Evolution of Phenolic Compounds and Astringency during Aging of Red Wine: Effect of Oxygen Exposure before and after Bottling. J. Agric. Food Chem..

[44] Karbowiak T., Crouvisier-Urion K., Lagorce A., Ballester J., Geoffroy A., Roullier-Gall C., Chanut J., Gougeon R.D., Schmitt-Kopplin P., Bellat J.-P. (2019). Wine Aging: A Bottleneck Story. npj Sci. Food.

[45] Oliveira V., Lopes P., Cabral M., Pereira H. (2013). Kinetics of Oxygen Ingress into Wine Bottles Closed with Natural Cork Stoppers of Different Qualities. Am. J. Enol. Vitic..

[46] Oliveira A.S., Furtado I., Bastos M.D.L., Guedes De Pinho P., Pinto J. (2020). The Influence of Different Closures on Volatile Composition of a White Wine. Food Packag. Shelf Life.

[47] Sun H., Ma Y., He Y., Qiao S., Yang X., Tittel F.K. (2019). Highly Sensitive Acetylene Detection Based on a Compact Multi-Pass Gas Cell and Optimized Wavelength Modulation Technique. Infrared Phys. Technol..

[48] Zhang D., Wei Z., Han Y., Duan Y., Shi B., Ma W. (2023). A Review on Wine Flavour Profiles Altered by Bottle Aging. Molecules.

[49] Vidal J.-C., Caillé S., Samson A., Salmon J.-M. (2017). Comparison of the Effect of 8 Closures in Controlled Industrial Conditions on the Shelf Life of a Red Wine. BIO Web Conf..

[50] Pons A., Lavigne V., Thibon C., Redon P., Loisel C., Dubourdieu D., Darriet P. (2021). Impact of Closure OTR on the Volatile Compound Composition and Oxidation Aroma Intensity of Sauvignon Blanc Wines during and after 10 Years of Bottle Storage. J. Agric. Food Chem..

[51] Ugliano M., Dieval J.-B., Siebert T.E., Kwiatkowski M., Aagaard O., Vidal S., Waters E.J. (2012). Oxygen Consumption and Development of Volatile Sulfur Compounds during Bottle Aging of Two Shiraz Wines. Influence of Pre- and Postbottling Controlled Oxygen Exposure. J. Agric. Food Chem..

[52] Dimkou E., Ugliano M., Dieval J.B., Vidal S., Aagaard O., Rauhut D., Jung R. (2011). Impact of Headspace Oxygen and Closure on Sulfur Dioxide, Color, and Hydrogen Sulfide Levels in a Riesling Wine. Am. J. Enol. Vitic..

[53] Arapitsas P., Guella G., Mattivi F. (2018). The Impact of SO_2_ on Wine Flavanols and Indoles in Relation to Wine Style and Age. Sci. Rep..

[54] Gambuti A., Siani T., Picariello L., Rinaldi A., Lisanti M.T., Ugliano M., Dieval J.B., Moio L. (2017). Oxygen Exposure of Tannins-Rich Red Wines during Bottle Aging. Influence on Phenolics and Color, Astringency Markers and Sensory Attributes. Eur. Food Res. Technol..

[55] Ling M.-Q., Xie H., Hua Y.-B., Cai J., Li S.-Y., Lan Y.-B., Li R.-N., Duan C.-Q., Shi Y. (2019). Flavor Profile Evolution of Bottle Aged Rosé and White Wines Sealed with Different Closures. Molecules.

[56] Walther A.-K., Durner D., Fischer U. (2018). Impact of Temperature during Bulk Shipping on the Chemical Composition and Sensory Profile of a Chardonnay Wine. Am. J. Enol. Vitic..

[57] Giuffrida De Esteban M.L., Ubeda C., Heredia F.J., Catania A.A., Assof M.V., Fanzone M.L., Jofre V.P. (2019). Impact of Closure Type and Storage Temperature on Chemical and Sensory Composition of Malbec Wines (Mendoza, Argentina) during Aging in Bottle. Food Res. Int..

[58] Lan H., Li S., Yang J., Li J., Yuan C., Guo A. (2021). Effects of Light Exposure on Chemical and Sensory Properties of Storing Meili Rosé Wine in Colored Bottles. Food Chem..

[59] Pati S., Crupi P., Savastano M.L., Benucci I., Esti M. (2020). Evolution of Phenolic and Volatile Compounds during Bottle Storage of a White Wine without Added Sulfite. J. Sci. Food Agric..

[60] Grant-Preece P., Barril C., Schmidtke L.M., Scollary G.R., Clark A.C. (2017). Light-Induced Changes in Bottled White Wine and Underlying Photochemical Mechanisms. Crit. Rev. Food Sci. Nutr..

[61] Waterhouse A.L., Zhu J. (2020). A Quarter Century of Wine Pigment Discovery. J. Sci. Food Agric..

[62] Li Y., Kong D., Fu Y., Sussman M.R., Wu H. (2020). The Effect of Developmental and Environmental Factors on Secondary Metabolites in Medicinal Plants. Plant Physiol. Biochem..

[63] De Freitas V., Mateus N. (2011). Formation of Pyranoanthocyanins in Red Wines: A New and Diverse Class of Anthocyanin Derivatives. Anal. Bioanal. Chem..

[64] He F., Liang N.-N., Mu L., Pan Q.-H., Wang J., Reeves M.J., Duan C.-Q. (2012). Anthocyanins and Their Variation in Red Wines I. Monomeric Anthocyanins and Their Color Expression. Molecules.

[65] Bakker J., Timberlake C.F. (1997). Isolation, Identification, and Characterization of New Color-Stable Anthocyanins Occurring in Some Red Wines. J. Agric. Food Chem..

[66] Kanavouras A., Coutelieris F., Karanika E., Kotseridis Y., Kallithraka S. (2020). Color Change of Bottled White Wines as a Quality Indicator. OENO One.

[67] Kioroglou D., Mas A., Portillo M.C. (2020). Qualitative Factor-Based Comparison of NMR, Targeted and Untargeted GC-MS and LC-MS on the Metabolomic Profiles of Rioja and Priorat Red Wines. Foods.

[68] Bueno M., Marrufo-Curtido A., Carrascón V., Fernández-Zurbano P., Escudero A., Ferreira V. (2018). Formation and Accumulation of Acetaldehyde and Strecker Aldehydes during Red Wine Oxidation. Front. Chem..

[69] Strobl M. (2019). Red Wine Bottling and Packaging. Red Wine Technology.

[70] Mayr C.M., Capone D.L., Pardon K.H., Black C.A., Pomeroy D., Francis I.L. (2015). Quantitative Analysis by GC-MS/MS of 18 Aroma Compounds Related to Oxidative Off-Flavor in Wines. J. Agric. Food Chem..

[71] Rousserie P., Rabot A., Geny-Denis L. (2019). From Flavanols Biosynthesis to Wine Tannins: What Place for Grape Seeds?. J. Agric. Food Chem..

[72] Drinkine J., Lopes P., Kennedy J.A., Teissedre P.-L., Saucier C. (2007). Ethylidene-Bridged Flavan-3-Ols in Red Wine and Correlation with Wine Age. J. Agric. Food Chem..

[73] Smith P.A., McRae J.M., Bindon K.A. (2015). Impact of Winemaking Practices on the Concentration and Composition of Tannins in Red Wine: Impact of Winemaking Practices on Tannins. Aust. J. Grape Wine Res..

[74] Millet M., Poupard P., Guilois-Dubois S., Zanchi D., Guyot S. (2019). Self-Aggregation of Oxidized Procyanidins Contributes to the Formation of Heat-Reversible Haze in Apple-Based Liqueur Wine. Food Chem..

[75] Perestrelo R., Silva C., Gonçalves C., Castillo M., Câmara J.S. (2020). An Approach of the Madeira Wine Chemistry. Beverages.

[76] Cejudo-Bastante M.J., Hermosín-Gutiérrez I., Pérez-Coello M.S. (2011). Micro-Oxygenation and Oak Chip Treatments of Red Wines: Effects on Colour-Related Phenolics, Volatile Composition and Sensory Characteristics. Part I: Petit Verdot Wines. Food Chem..

[77] Sacks G.L., Gates M.J., Ferry F.X., Lavin E.H., Kurtz A.J., Acree T.E. (2012). Sensory Threshold of 1,1,6-Trimethyl-1,2-Dihydronaphthalene (TDN) and Concentrations in Young Riesling and Non-Riesling Wines. J. Agric. Food Chem..

[78] Collin S., Nizet S., Claeys Bouuaert T., Despatures P.-M. (2012). Main Odorants in Jura Flor-Sherry Wines. Relative Contributions of Sotolon, Abhexon, and Theaspirane-Derived Compounds. J. Agric. Food Chem..

[79] Sáenz-Navajas M.-P., Avizcuri J.-M., Ballester J., Fernández-Zurbano P., Ferreira V., Peyron D., Valentin D. (2015). Sensory-Active Compounds Influencing Wine Experts’ and Consumers’ Perception of Red Wine Intrinsic Quality. LWT—Food Sci. Technol..

[80] Vázquez-Pateiro I., Arias-González U., Mirás-Avalos J.M., Falqué E. (2020). Evolution of the Aroma of Treixadura Wines during Bottle Aging. Foods.

[81] Yang Y., Jin G.-J., Wang X.-J., Kong C.-L., Liu J., Tao Y.-S. (2019). Chemical Profiles and Aroma Contribution of Terpene Compounds in Meili (*Vitis vinifera* L.) Grape and Wine. Food Chem..

[82] Roland A., Schneider R., Razungles A., Cavelier F. (2011). Varietal Thiols in Wine: Discovery, Analysis and Applications. Chem. Rev..

[83] Tominaga T., Dubourdieu D. (2006). A Novel Method for Quantification of 2-Methyl-3-Furanthiol and 2-Furanmethanethiol in Wines Made from Vitis Vinifera Grape Varieties. J. Agric. Food Chem..

[84] Tominaga T., Guimbertau G., Dubourdieu D. (2003). Role of Certain Volatile Thiols in the Bouquet of Aged Champagne Wines. J. Agric. Food Chem..

[85] Bührle F., Gohl A., Weber F. (2017). Impact of Xanthylium Derivatives on the Color of White Wine. Molecules.

[86] Vallverdú-Queralt A., Meudec E., Eder M., Lamuela-Raventos R.M., Sommerer N., Cheynier V. (2017). The Hidden Face of Wine Polyphenol Polymerization Highlighted by High-Resolution Mass Spectrometry. ChemistryOpen.

[87] He Y., Wang X., Li P., Lv Y., Nan H., Wen L., Wang Z. (2023). Research Progress of Wine Aroma Components: A Critical Review. Food Chem..

[88] He J., Zhou Q., Peck J., Soles R., Qian M.C. (2013). The Effect of Wine Closures on Volatile Sulfur and Other Compounds during Post-bottle Aging. Flavour Fragr. J..

[89] Ferreira V., Bueno M., Franco-Luesma E., Culleré L., Fernández-Zurbano P. (2014). Key Changes in Wine Aroma Active Compounds during Bottle Storage of Spanish Red Wines under Different Oxygen Levels. J. Agric. Food Chem..

[90] Harutyunyan M., Malfeito-Ferreira M. (2022). Historical and Heritage Sustainability for the Revival of Ancient Wine-Making Techniques and Wine Styles. Beverages.

[91] Lopes P., Silva M.A., Pons A., Tominaga T., Lavigne V., Saucier C., Darriet P., Teissedre P.-L., Dubourdieu D. (2009). Impact of Oxygen Dissolved at Bottling and Transmitted through Closures on the Composition and Sensory Properties of a Sauvignon Blanc Wine during Bottle Storage. J. Agric. Food Chem..

[92] Cejudo-Bastante M.J., Hermosín-Gutiérrez I., Pérez-Coello M.S. (2013). Accelerated Aging against Conventional Storage: Effects on the Volatile Composition of Chardonnay White Wines. J. Food Sci..

[93] Gómez-Plaza E., Cano-López M. (2011). A Review on Micro-Oxygenation of Red Wines: Claims, Benefits and the Underlying Chemistry. Food Chem..

[94] Lisanti M.T., Capuano R., Moio L., Gambuti A. (2021). Wood Powders of Different Botanical Origin as an Alternative to Barrel Aging for Red Wine. Eur. Food Res. Technol..

[95] Yang Y., Deed R.C., Araujo L.D., Waterhouse A.L., Kilmartin P.A. (2022). Effect of Microoxygenation Applied before and after Malolactic Fermentation on Monomeric Phenolics and Tannin Composition of Pinot Noir Wine. Aust. J. Grape Wine Res..

[96] Renner H., Richling E., Durner D. (2023). Investigation of Molecular Changes in Wine during Storage in Refrigerated Cabinets. Lebensmittelchemie.

[97] Celotti E., Stante S., Ferraretto P., Román T., Nicolini G., Natolino A. (2020). High Power Ultrasound Treatments of Red Young Wines: Effect on Anthocyanins and Phenolic Stability Indices. Foods.

[98] Yıldırım H.K., Dündar E. (2017). New Techniques for Wine Aging. BIO Web Conf..

[99] Wu Z., Li X., Zeng Y., Cai D., Teng Z., Wu Q., Sun J., Bai W. (2022). Color Stability Enhancement and Antioxidation Improvement of Sanhua Plum Wine under Circulating Ultrasound. Foods.

[100] Blake A., Kotseridis Y., Brindle I.D., Inglis D., Pickering G.J. (2010). Effect of Light and Temperature on 3-Alkyl-2-Methoxypyrazine Concentration and Other Impact Odourants of Riesling and Cabernet Franc Wine during Bottle Aging. Food Chem..

[101] Cvetkova S., Hirt B., Stahl M., Scharfenberger-Schmeer M., Durner D. (2023). UV-C Treatment: A Non-Thermal Inactivation Method for Microbiological Stabilisation of Must and Wine. BIO Web Conf..

[102] Lezaeta A., Bordeu E., Agosin E., Pérez-Correa J.R., Varela P. (2018). White Wines Aroma Recovery and Enrichment: Sensory-Led Aroma Selection and Consumer Perception. Food Res. Int..

[103] Ding Q., Wang H., Zhou Y., Zhang Z., Luo Y., Wu Z., Yang L., Xie R., Yang B., Tao K. (2025). Self-Powered Switchable Gas-Humidity Difunctional Flexible Chemosensors Based on Smart Adaptable Hydrogel. Adv. Mater..

[104] Birkić N., Ožbolt E., Reynolds C.A., Pavlešić T., Lučin I., Andabaka Ž., Saftić Martinović L. (2024). Maturation of Wine in Underwater Springs as a Novel Wine Production Process. Eur. Food Res. Technol..

[105] Balivo A., D’Auria G., Ferranti P., Cepollaro A., Velotto S., Sacchi R., Genovese A. (2025). Impact of Underwater Aging on the Volatile and Phenolic Compounds of Campania Wine-Based Liqueurs “Elixir Falernum”. Beverages.

[106] Mercanti N., Pieracci Y., Macaluso M., Fedel M., Brazzarola F., Palla F., Verdini P.G., Zinnai A. (2024). Exploring Red Wine Aging: Comparative Analysis of Cellar and Sea Underwater Aging on Chemical Composition and Quality. Foods.

[107] Maioli F., Picchi M., Bandinelli A., Colavolpe G., Kottakhs E., Canuti V. (2025). Effect of Underwater Aging Treatment on Wine Quality: A Preliminary Study. Eur. Food Res. Technol..

[108] Martínez-Moreno A., Martínez-Pérez P., Bautista-Ortín A.B., Gómez-Plaza E. (2023). Use of Unripe Grape Wine as a Tool for Reducing Alcohol Content and Improving the Quality and Oenological Characteristics of Red Wines. OENO One.

[109] Cosme F., Morais R., Peres E., Cunha J.B., Fraga I., Milheiro J., Filipe-Ribeiro L., Mendes J., Nunes F.M. (2019). Precision Enology in Tawny Port Wine Aging Process: Monitoring Barrel to Barrel Variation in Oxygen, Temperature and Redox Potential. BIO Web Conf..

[110] Mulero-Cerezo J., Tuñón-Molina A., Cano-Vicent A., Pérez-Colomer L., Martí M., Serrano-Aroca Á. (2022). Probiotic Rosé Wine Made with *Saccharomyces cerevisiae* var. *Boulardii*. Preprints.

[111] Ellis D.J., Kerr E.D., Schenk G., Schulz B.L. (2022). Metabolomics of Non-Saccharomyces Yeasts in Fermented Beverages. Beverages.

[112] Zhao H., Liu S., Zhu L., Wang Y. (2025). Microorganisms: The Key Regulators of Wine Quality. Compr. Rev. Food Sci. Food Saf..

[113] Silva P. (2024). Low-Alcohol and Nonalcoholic Wines: From Production to Cardiovascular Health, along with Their Economic Effects. Beverages.

